# Exploring an Intermediate Colorectal Cancer Screening Test Based on Stool Proteomics and Machine Learning for Optimizing the Selection of Patients for Colonoscopy Identified From FIT

**DOI:** 10.1016/j.mcpro.2026.101534

**Published:** 2026-02-19

**Authors:** David Gagné, Elmira Shajari, Mandy Malick, Patricia Roy, Jean-François Noël, Hugo Gagnon, Marie A. Brunet, Julie C. Carrier, François-Michel Boisvert, Jean-François Beaulieu

**Affiliations:** 1 Laboratory of Intestinal Physiopathology, Faculty of Medicine and Health Sciences, Université de Sherbrooke, Sherbrooke, Quebec, Canada; 2 Centre de Recherche du Centre Hospitalier Universitaire de Sherbrooke, Sherbrooke, Quebec, Canada; 3 Department of Immunology and Cell Biology, Faculty of Medicine and Health Sciences, Université de Sherbrooke, Sherbrooke, Quebec, Canada; 4 Department of Medicine, Faculty of Medicine and Health Sciences, Université de Sherbrooke, Sherbrooke, Quebec, Canada; 5 Allumiqs Solutions, Sherbrooke, Quebec, Canada; 6 Department of Pediatrics, Faculty of Medicine and Health Sciences, Université de Sherbrooke, Sherbrooke, Quebec, Canada

**Keywords:** colorectal cancer screening, fecal immunochemical test, proteomics, false positive

## Abstract

The fecal immunochemical test (FIT) for detecting fecal occult blood, used alone or in combination with other stool biomarkers, has been demonstrated to be effective in the context of colorectal cancer (CRC) screening programs. However, FIT yields a significant proportion of false positives leading to unnecessary colonoscopies. In this study, we have investigated whether leftover FIT stool samples could be repurposed for proteomics analysis as a triage step for patients before recommending colonoscopy. High-throughput mass spectrometry analyses on a set of 141 FIT-positive samples (50 controls with no lesion, 45 with advanced adenomas and 46 with CRC) in combination with machine learning tools were used. Results showed that with a specificity ≥90%, a large proportion of the false FIT positives could be identified thus providing an efficient strategy for reducing unnecessary colonoscopies. Furthermore, CRC cases were also precisely predicted to be true positives, thus providing an approach for prioritizing patients for colonoscopy. In conclusion, this study demonstrates the feasibility of using proteomics for analysis of leftover FIT stool samples as an intermediate step to triage patients selected for colonoscopy in CRC screening programs.

## Highlights

• Proteomic analysis of leftover FIT samples was explored.
• High-throughput mass spectrometry and machine learning tools were used.
• Proteomic signatures identify most of the false FIT-positive patients.
• Proteomic signatures predicted patients with colorectal cancer (CRC).
• This study provided the basis for further improving CRC screening.

## Graphical Abstract

**Figure:**
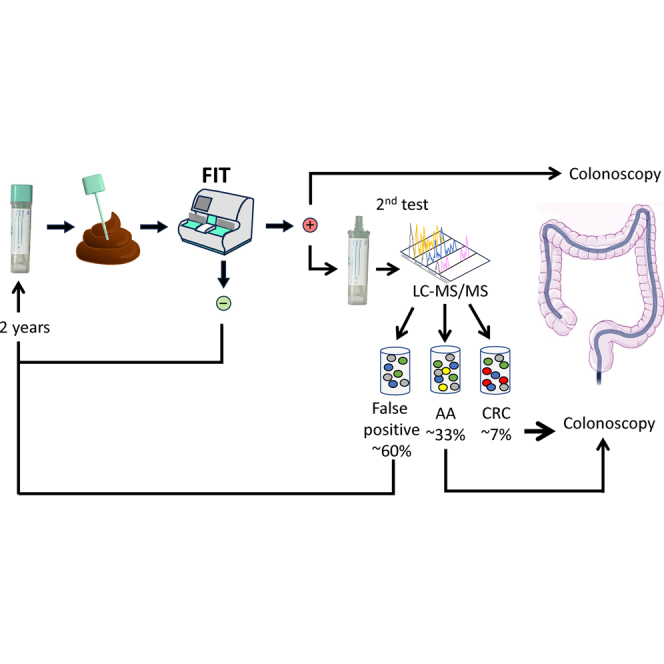

## In Brief

This study by Gagné *et al*. has tested the feasibility of using proteomics for the analysis of leftovers of the fecal immunochemical test (FIT) stool samples as an intermediate step to triage patients selected for colonoscopy in the colorectal cancer (CRC) screening programs. Analyses on a set of 141 FIT-positive samples in combination with machine learning tools showed that a large proportion of the false FIT positives could be identified, thus providing an efficient strategy for reducing unnecessary colonoscopies.

Colorectal cancer (CRC) is among the deadliest cancer types (1) but at the same time one of the few for which screening has been proven to reduce mortality in average-risk individuals (2). Indeed, while the 5-year survival rate of CRC is near 90% for individuals with circumscribed lesions, it is only 14% for those having metastases (3). Early detection is thus a crucial factor for improving survival from CRC (4, 5). In this context, it is notable that implementation of screening programs has led to a reduction in incidence of CRC and related mortality (6, 7). However, adherence to CRC screening guidelines appears to be well below the optimal target of 80% proposed by the American Cancer Society (8) as it has been reported in 2021 to be only at 59% (9).

The gold standard for the detection of CRC as well as advanced adenomas (AAs), the CRC precursors (10, 11), is colonoscopy. However, discomfort and unpleasant preparation procedures affect compliance (12). The risk of complications, cost, and access are other constraints limiting the universality of colonoscopy as a first-step screening option (13, 14). That has led to the development and application of noninvasive methods to screen asymptomatic eligible persons to select those with a positive test for follow-up with colonoscopy (15). Guaiac-based tests were the first to be implemented for this purpose but were then replaced by an improved immunological version of fecal occult blood testing also referred to as the fecal immunochemical test (FIT), which detects human hemoglobin (15). FIT has been used with some success (2) although poor precursor lesion detection rates (66–80% sensitivity for CRC but only 10–28% for AA), albeit an excellent specificity (93–95%), is one of its limitations (4, 16, 17, 18).

A blood-independent CRC feature that was then considered for stool-based testing strategies is the high rate of tumor cell exfoliation into the colon-rectal lumen (19, 20, 21, 22), which led to the testing of new screening modalities. For instance, the detection of specific DNA aberrations from the CRC cells shed into the stools in combination with FIT, for which kits are commercially available (https://coloalert.com/; https://www.cologuard.com/), was shown to result in an improvement of sensitivity for both CRC and AA detection compared to FIT alone, although achieved through a reduction of specificity thus generating two to three times more false positives than FIT alone (23, 24, 25). New versions of this approach are under study. For instance, the BLUE-C study which consists of updated DNA markers from the first DeeP-C study (23) showed improved sensitivity for CRC at 94% (but not for AA which remained around 43%) and higher specificity at 90.6% (26). Inclusion of other types of nucleic acid markers (27, 28) may become an additional source of improvement for CRC and AA sensitivity. However, one limitation to these new approaches remains the relatively high number of false positives which leads to a significant number of unnecessary colonoscopies (29). It is also noteworthy that a significant decrease in specificity with increasing age was noted in the BLUE-C study (26). In a screening test, 5 to 9% of false positives represent a significant burden to both financial and clinical resources. Indeed, there is a consensus that 45 to 70% of the colonoscopies performed in the context of a positive FIT corresponds to non-neoplastic or negative findings (30). The situation with the mtDNA/FIT test appears similar. For instance, in the BLUE-C study, among participants with a positive test, CRC was detected in 3.4% and AA in 34.3% (26). In this context, the introduction of complementary screening tests using biomarker detection directly from the leftover FIT stool samples to improve the triage of individuals for colonoscopy appears to be a promising approach.

In this study, we have evaluated the potential of proteomics-based stool sample analyses as a triage tool for patients invited to pass a colonoscopy after having tested positive to a FIT. Our laboratory has previously shown the potential of combining high-throughput mass spectrometry (MS) analyses of stool samples and machine learning (ML) for successfully predicting necrotizing enterocolitis development in premature neonates (31) as well as classifying patients with inflammatory bowel diseases (32).

## Experimental Procedures

### Sample Collection and Ethics Approval

The samples were obtained from the Clinical Biochemistry Laboratory of the Centre Intégré de Santé et de Services Sociaux (CIUSSS) de l’Estrie-Centre Hospitalier Universitaire de Sherbrooke (CHUS) as part of the Quebec FIT screening program recommended to people between the ages of 50 and 74 and above. Fecal samples (approximately 10 μg of stool) were self-collected by each volunteer using the OC-Auto FIT test kit Polymedco (Somagen Diagnostics). Based on the recommendation of the Institut National d'Excellence en Santé et Services Sociaux, the detection threshold for positive test results has been set at 175 ng/ml for Québec (33). The research protocol for accessing the residual stool samples from patients that have been tested for FIT includes a reverse consent procedure for using residual stool samples and access to the related clinical data on the Ariane network for diagnosis. The study was conducted in accordance with the Declaration of Helsinki. The protocol has been approved by the Research Ethics Committee of the CIUSSS de l'Estrie-CHUS (Protocol # 2021-4074; last date of approval February 9, 2025). Inclusion and exclusion criteria: Inclusion criteria were a positive FIT (beyond the 175 ng/ml threshold), consent for inclusion, recommendation for colonoscopy. Exclusion criteria included lack of availability of corresponding colonoscopy and/or pathology reports (lack of or incomplete colonoscopy); previous history of colorectal lesion.

Remaining FIT samples were kept frozen at −80 ^o^C until confirmation of a FIT-positive result and the medical diagnosis registered to the patient’s medical record validated by a gastroenterologist: Gr1 (G1) for positive FIT results diagnosed by colonoscopy as negative for AA (defined as larger than 1.0 cm and/or serrated and/or displaying high grade dysplasia) and CRC (stages I–III); Gr2 (G2) for positive for FIT results and positive diagnosis for AA and no CRC; Gr3 (G3) for positive FIT results and positive diagnosis for CRC. Demographic and clinical characteristics of the patients are provided in Supplemental Table S1.

### Sample Preparation

Samples thawed on ice were extracted from the FIT test tubes and centrifuged for 5 min at 5000×*g* before being aliquoted and frozen at −80 °C. For LC−MS/MS analyses, 30 μg of proteins were taken from the supernatant and brought to 100 μl with 50 mM Tris buffer at pH 8.5 and subjected to reduction in 10 mM DTT for 10 min at 65 °C and alkylation in 15 mM iodoacetamide for 30 min at room temperature before quenching (in 10 mM DTT). Proteins were precipitated in acetone for 1 h at −80 °C, followed by centrifugation at 16,000×*g* for 15 min and washing with cold methanol. Protein digestion was carried out overnight in 100 μl of 50 mM Tris buffer (pH 8.0) using a proteolytic mix of Trypsin/Lys-C, MS Grade (Promega; 30:1 w/w protein/protease ratio), with the addition of 0.08 μg of Digestif Labeled 13C15N (Promise Advanced Proteomics) for internal control. The digestion was halted with 2% formic acid. Peptides were purified using a reverse-phase Strata-X polymeric solid-phase extraction sorbent column (Phenomenex), according to the manufacturer’s instructions. The recovered peptides were dried under nitrogen flow and stored at 4 °C until being resuspended in 25 μl of mobile phase solvent A (0.2% v/v formic acid, 3% v/v DMSO in water) before LC−MS/MS analysis.

### LC−MS/MS Settings

Data acquisitions were conducted at the proteomics facilities of Allumiqs Solutions (Sherbrooke) as described previously (31, 32) on a MS system (ABSciex TripleTOF 6600, ABSciex), equipped with an electrospray interface featuring a 25 μm ID capillary and coupled to an Eksigent UHPLC system (Eksigent). Sample separation was achieved using a reverse-phase Kinetex XB column (0.3 mm ID, 2.6 μm particles, 150 mm length; Phenomenex) maintained at 60 °C. The liquid chromatography (LC) gradient utilized two mobile phases: solvent A (0.2% v/v formic acid, 3% DMSO v/v in water) and solvent B (0.2% v/v formic acid, 3% DMSO v/v in ethanol). The source voltage was set at 5.5 kV and maintained at 325 °C, with the curtain gas set at 35 psi, gas 1 at 27 psi, and gas 2 at 10 psi. Analyst TF 1.8 software (https://sciex.com/cr/landing-pages/analyst-tf-software-18, ABSciex) was used to control the instrument settings. Various parameters were evaluated using the Sequential Window Acquisition of All Theoretical Mass Spectra (SWATH) Variable Window Calculator (ABSciex) to optimize data-independent acquisition (DIA) on a 30-min LC gradient. This included evaluating the number, width, and distribution of SWATH variable isolation windows based on *m/z* intensity distribution for the MS1 range of 350 − 1250 *m/z*. The optimal method provided at least 6 MS2 data points per peak while maximizing the number of quantifiable proteins and peptides, using 72 minimally overlapping isolation windows (1 *m/z*) acquired without a staggered scheme.

### Spectral Library and Label-free Quantification

To increase fecal proteome coverage, MS data files from the FIT samples were combined with 10 additional MS files acquired from fecal samples under identical LC–MS/MS conditions (32). Two complementary strategies were employed to construct a spectral library: (1) A spectrum-centric approach based on detection of pseudo–MS/MS spectra (34) with the FragPipe computational platform (v19.1) (35), and (2) AI-assisted spectra extraction using a deep learning, predicted *in silico* library with DIA-NN (v1.8.1) (36).

Raw .wiff files (ABSciex) were converted to mzML format using ProteoWizard with peak picking filter for signal extraction and DIA-Umpire for pseudo-MS/MS generation (34, 37). Subsequent steps, including thresholding, deisotoping, charge state and retention time assignment, and false discovery rate (FDR) estimation were performed by downstream software. DIA-NN has been shown to exert stringent FDR control in diverse settings, including with external (non–DIA-NN) library and mixed-species datasets (38), and was therefore used in a multistage FDR control workflow for refinement of the FragPipe-derived spectral library and for the final precursor-level and protein-level quantification.

The pseudo-MS/MS files were processed with FragPipe, which integrates MSFragger, Philosopher, Percolator, MSBooster, and EasyPQP (35, 39, 40, 41). FragPipe was used for peptide identification, PSM validation, protein inference, FDR control, and spectral library generation. Search settings included precursor and fragment mass tolerances of 20 ppm, peptide lengths of 6 to 42 amino acids, strict trypsin (K/R) digestion with up to two missed cleavages, methionine oxidation, N-terminal acetylation, and serine/threonine phosphorylation as variable modifications, and cysteine carbamidomethylation as a fixed modification. Relaxed FDR thresholds were set at 5% for proteins and 1% for peptides. In total, this spectral-library search space encompassed 157,260 protein sequences. This included the reviewed human proteome (UP000005640; 42,413 entries including isoforms and common contaminants, covering 20,389 *Homo sapiens* proteins; downloaded 13 February 2023 from UniProt.org). To account for potential exogenous proteins originating from animal-derived dietary components, we also included the unreviewed proteomes of bovine (*Bos taurus*, UP000009136; 37,519 entries), pig (*Sus scrofa*, UP000008227; 49,793 entries), and chicken (*Gallus gallus*, UP000000539; 27,535 entries). This initial Fragpipe library was subsequently refined using DIA-NN (v1.8.1) in match-between-runs (MBRs) mode, which creates a new optimized library from the DIA-SWATH data (peak picked mzML) [6], with reannotation based on the same proteome files, protein inference performed at the gene level (species-specific), and a 1% PSM FDR threshold applied at the precursor level.

A second library was also generated using DIA-NN’s library-free mode (36), with settings equivalent to those described above. For this approach, we used a list of 3790 human protein IDs previously detected at least once in fecal samples across independent projects (31, 32), including those identified by FragPipe in the first library. These IDs were converted into a FASTA file (8223 entries, including isoforms) using UniProt’s ID mapping tool. The FASTA database was then used by DIA-NN to generate an *in silico*–predicted spectral library and extract corresponding DIA spectra.

The two libraries were subsequently integrated and filtered to remove duplicate precursors. Preliminary DIA-NN analysis revealed exogenous biological contaminants in the stabilizing buffer of blank FIT vials. The corresponding contaminants precursors were removed from the spectral library. Then, label-free quantification was performed in DIA-NN using MBR mode with Robust LC (high-accuracy) quantification, protein inference at the gene level (species-specific), and a 1% PSM FDR threshold applied at the precursor level. The resulting MBR-optimized spectral library contained 10,946 precursors derived from 9715 unique peptide sequences, which were inferred to 2354 unique proteins or protein groups.

The protein matrices output with quantified proteins was generated using DIA-NN’s (v1.8.1) MaxLFQ approach, with 1% FDR, applied to global q values at the protein-group level and to both global and run-specific q values at the precursor level, with an additional 1% FDR filter applied to the matrices. In total, 1979 proteins or protein groups were detected with at least one quantified value: 1383 exclusively human; 380 human proteins co-inferred with other species (bovine, pig, and/or chicken); and 217 originating exclusively from nonhuman species.

## Experimental Design and Statistical Rationale

### Study Overview

**Fig. 1:**
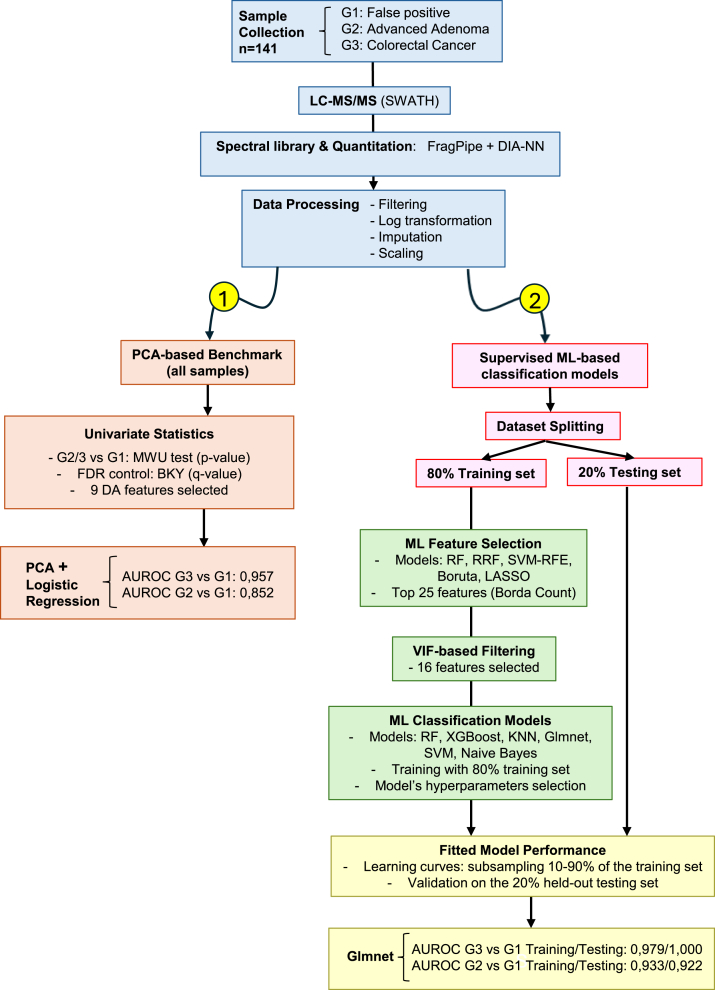
**Flowchart of the pipeline for biomarker discovery using two strategies for discriminating between samples assigned to AA and CRC diagnosis from false-positive FIT results.** (1) principal component analysis (PCA)-based benchmark model and (2) supervised ML–based classification models. AA, advanced adenoma; BKY, Benjamini–Krieger–Yekutieli two-stage step-up procedure; CRC, colorectal cancer; DA, differentially abundant; FIT, fecal immunochemical test; Glmnet, generalized linear model with elastic net regularization; KNN, K-nearest neighbors; Lasso, least absolute shrinkage and selection operator; ML, machine learning; MWU, Mann–Whitney *U* test; PCA, principal component analysis; RF, Random Forest; RFE, Recursive Feature Elimination; RRF, Regularized Random Forest; SVM, Support Vector Machine; SWATH, Sequential Window Acquisition of All Theoretical Fragment-ion Spectra; VIF, variance inflation factor; XGBoost, Extreme Gradient Boosting.

This study was designed to identify stool proteomic signatures in residual FIT samples capable of discriminating samples assigned to the AA (G2) and CRC (G3) diagnostic groups from false-positive FIT results (G1, no AA/CRC). The main steps of the experimental design, including sample collection, data processing, feature selection, and model development, are summarized in Figure 1, which presents the overall workflow for biomarker discovery and sample classification.

### LC−MS/MS Data Acquisition and Integration

A total of 141 FIT samples (G1: 50, G2: 45, G3: 46) were included in the proteomics analysis. Sample preparation and LC–MS/MS acquisitions were separated across four balanced batches, each containing approximately equal numbers of samples from G1, G2, and G3. Each batch also included three pooled QC samples to monitor LC–MS/MS signal stability and one blank FIT sample (buffer without fecal material) to monitor potential exogenous biological contaminants. Digestif Labeled 13C15N (Promise Advanced Proteomics) was spiked into each sample to monitor efficacy of the trypsin digestion (see Sample Preparation section above for details).

A DIA–SWATH method with a 30-min LC gradient acquired non-fractionated MS1 (350–1250 *m/z*) and MS2 (100–1800 *m/z*) data using 72 optimized, overlapping 1 *m/z*-wide isolation windows (total cycle time: 1.54 s) (see LC−MS/MS Settings and Acquisition section above for details). To minimize the impact of potential intra-batch signal variation that could introduce group-specific bias, samples were randomized prior to data acquisition using simple randomization of the full sample list injection order (uniform random permutation without replacement-equal probability of occupying any position within the batch). QC and blank samples were subsequently inserted at fixed intervals.

A spectral library was built from all the DIA-SWATH data and from 10 additional MS files acquired from fecal samples under identical LC–MS/MS conditions, for a total of 163 samples (141 individual FIT, 12 QC-pools and 10 fecals). The library construction, peptide detection, protein inference, peak integration and label-free quantification were performed as described in the Spectral Library and Label-Free Quantification section above.

### Data Processing

The dataset was filtered to remove proteins with fewer than 50% quantified values across the 141 FIT samples, a threshold chosen to accommodate proteins that may be preferentially expressed in specific groups. In addition, only proteins or protein groups inferred to human, either exclusively (n = 307) or with another dietary species (n = 27) were retained. After filtering, 334 proteins remained, with an overall quantification rate of 78%.

Prior to statistical analysis, a five-step processing pipeline established in a prior proteomics study (32) was implemented in R (v4.2.3) (42) within the RStudio environment (v2024.04.02) (43). This pipeline was selected to ensure that the dataset met key statistical assumptions, including variance stabilization, comparability across samples and proteins, and minimal technical bias.

Steps 1 and 2 consisted of log2 transformation (Base R package, v4.2.3) (42) and quantile normalization (preprocessCore package, v1.60.2) (44), respectively, to stabilize variance and align intensity distributions. In step 3, batch effects were evaluated and corrected. Linear modeling with empirical Bayes moderation (limma package, v3.64.3) (45) indicated that approximately 25% of proteins were significantly influenced by batch. Batch correction was performed using ComBat (sva package, v3.46.0) (46), an empirical Bayes–based method commonly used in proteomics because it effectively reduces technical variability when batch sizes are limited (47, 48). In step 4, missing values were imputed using k-nearest neighbors across samples (KNNs, k = 10; impute package, v1.72.3) (49). This approach preserves local correlation structure among similar samples and provides biologically plausible estimates for missing values. Finally, in step 5, z-score scaling (Base R package, v4.2.3) was applied to center each protein standardize variance across variables, ensuring equal contribution to downstream analyses and modeling.

### Differential Abundance Analysis

Differentially abundant proteins (G2/3 *versus* G1) in the processed complete dataset (141 samples) were identified using the Mann–Whitney *U* test (GraphPad Prism, v9.3.1; GraphPad Software; https://www.graphpad.com/) (50). This nonparametric, rank-based test is suitable for comparing unmatched groups in the presence of moderate class imbalance and does not require assumptions about the underlying distributions. Multiple testing correction was applied using the Benjamini–Krieger–Yekutieli two-stage step-up procedure (as recommended in GraphPad Prism) to control the FDR (51), yielding q values. Proteins with q values <0.05 were considered statistically significant.

### Supervised ML Feature Selection

The processed dataset was partitioned into an 80% training set (113 samples: used here for ML feature selection, and later for ML classification model training and hybrid learning curve generation) and a 20% held-out testing set (28 samples: kept unseen until final ML classification models validation) using stratified sampling (caret package, v6.0.94) (52) in R to preserve class proportions. Feature selection was performed on the 80% training set using five supervised ML algorithms: Random Forest (RF; randomForest package, v4.7.1.2) (53), Regularized Random Forest (RRF; RRF package, v1.9.4) (54), Support Vector Machine with Recursive Feature Elimination (SVM-RFE; caret package, v6.0.94; method = “svmRadial”), Boruta (Boruta package, v8.0.0) (55), and Lasso (glmnet package, v4.1.8) (56).

To minimize the instability inherent to individual algorithms, each method was run 50 times with different random seeds (set.seed) in R (Base R package, v4.2.3). Feature rankings (or absolute coefficient for Lasso) were aggregated using a Borda count–based rank integration (R custom implementation) to obtain a Top-25 feature list for each algorithm (57). The five algorithm-specific lists were then combined using the same Borda count strategy to generate the final Top-25 consensus feature set, representing the proteins with the highest cumulative importance across all methods. By integrating five different ML algorithms through Borda count aggregation, this approach reduces model-specific bias and enhances the robustness of selected features.

### Feature Validation and Filtering

Although DIA-NN (v1.8.1) implements advanced signal discrimination and interference-corrected quantification strategies (36), automated processing can be challenged by complex samples containing peptides from multiple sources (human, feed, microbiome, etc.). To ensure that features identified as statistically significant and selected by ML models were quantified reliably, these candidates were visually inspected in Skyline (MacCoss Lab, University of Washington) (58). Features with poor spectral reliability (e.g., very weak, noisy or inconsistent signals, potential interference) were excluded from downstream analyses.

Highly correlated features introduce multicollinearity, which negatively affects the performance and interpretability of regression and ML classification models (59). To address multicollinearity, variance inflation factors (VIFs) were calculated in R using the car package (v3.1.3) (60). For features selected by ML models with VIF ≥10, one representative variable was retained and the others removed, reducing collinearity before classification analyses.

Outlier detection was performed using the ROUT method (Q = 1%; GraphPad Prism) within each class (G1 and G2/G3) (61), as this method improves detection when multiple outliers may be present.

### Classification Models

Two strategies were employed to evaluate the ability to discriminate between samples assigned to AA and CRC diagnostic groups from false-positive FIT results (G1 *versus* G2/3): (1) a principal component analysis (PCA)-based benchmark model and (2) supervised ML-based classification models.

The simpler PCA-based model applied unsupervised dimensionality reduction using PCA (41), followed by binary multivariable logistic regression to estimate group membership probabilities. As this model was constructed for exploratory and benchmarking purposes, processed quantitative values of differentially abundant proteins (q value <0.05) from all samples in the dataset were used, without splitting into training and testing sets and further validation. The number of significant principal components (PCs) was determined via parallel analysis (62), retaining PCs whose eigenvalues exceeding the 95th percentile of those from random datasets generated by Monte Carlo simulation (GraphPad Prism). This approach provides a statistically validated criterion compared with arbitrary variance cutoffs (e.g., Kaiser's rule). Sample scores on the retained PCs were then used as predictors in the logistic regression model, and binary performance metrics (63)—sensitivity (true-positive rate), specificity (true negative rate), balanced accuracy (mean of sensitivity and specificity), and the area under the receiver operating characteristic curve (AUROC)—were calculated from predicted class probabilities.

In contrast, all supervised ML classification models were trained exclusively on the 80% training set, consistent with the ML feature-selection procedures described above. Six algorithms were implemented in R within the *caret* (v6.0.94) framework (52): RF (ranger package, v0.16.0; method = "ranger") (64), penalized logistic regression (Lasso, Ridge, and Elastic Net; glmnet, v4.1.8; method = "glmnet") (56), KNNs (method = “knn”), Extreme Gradient Boosting (xgboost package, v1.7.8.1, method = "xgbTree") (65), Support Vector Machine (SVM, method = "svmPoly"), and Naïve Bayes (method = "nb").

Model training used 10-fold cross-validation repeated three times (method = “repeatedcv”), with stratification applied to account for class imbalance. Hyperparameters were tuned using predefined search grids (Base R expand.grid; see Supplemental Table S2). AUROC was defined as the *a priori* optimization metric for hyperparameter selection due to its robustness to class imbalance and threshold-independent evaluation of classifier performance (66). Additional binary performance metrics were obtained from caret-based confusion matrices.

### Model Evaluation and Interpretability

All model evaluation and interpretability analyses were conducted using fixed optimal hyperparameter configurations previously identified during training on the full 80% training set.

A hybrid learning curve approach was used to evaluate model learning behavior and stability. This approach extends standard learning curve analysis (67, 68) by applying repeated stratified hold-out evaluation (also known as Monte Carlo cross-validation) across increasing training sizes, thereby providing robust estimates of learning dynamics and performance variability. For each model, stratified random sampling was used to generate training subsets comprising 10% to 90% of the full 80% training dataset, with 10 repeats per training size. At each iteration, models were refitted on the corresponding training subset using the fixed optimal hyperparameters and evaluated on the corresponding internal hold-out subsets (complementary 90%–10%).

Model performance was further evaluated by comparing each fitted model’s training AUROC with its mean cross-validated (CV) AUROC to detect potential overfitting (69). Models showing favorable learning dynamics and stability, with minimal evidence of overfitting, were selected for evaluation on the independent 20% held-out testing set to obtain unbiased estimates of generalization performance.

To enhance model interpretability, SHapley Additive exPlanations (SHAP) values were calculated in R using the kernelshap package (v0.7.0) (70) for the selected fixed classifier to quantify feature contributions and provide insights into feature importance and model decision-making.

### Plotting and Visualization

Several visualizations were generated to summarize key results. The volcano plot of differentially abundant proteins was produced in GraphPad Prism. The 3D PCA plot for the PCA-based model was created in R using the plotly package (v4.10.4) (71). Feature-importance and Top-25 feature histograms, feature-selection stability plots, and learning curves were generated using the ggplot2 package (v3.5.1) (72). The Venn diagram illustrating top 25 features selection consistency was created with the VennDiagram package (v1.7.3) (73). Receiver operating characteristic (ROC) curves for both training and testing datasets were generated from class-probability outputs using the pROC package (v1.18.5) (74). SHAP value beeswarm plots were produced using the shapviz package (v0.9.6) (75).

## Results

### PCA-Based Model

**Fig. 2:**
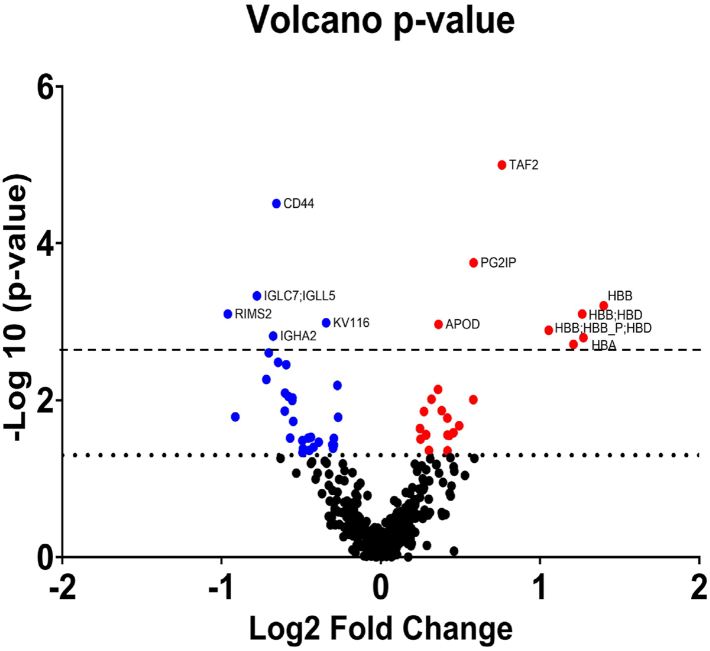
**Volcano plot of differentially abundant proteins between samples from the combined group of advanced adenomas and colorectal cancers (G2/G3) *versus* the control group (G1)**. The *x*-axis represents the log_2_ fold change in mean protein abundance between the two groups, while the *y*-axis shows the –log_10_ of the *p* value. Among the 334 proteins consistently detected across samples, 57 were initially identified as either decreased (*blue dots*) or increased (*red dots*) in G2/G3 samples, based on *p* ≤ 0.05 (above the *dotted line*: –log_10_*p* ≥ 1.3) from Mann–Whitney *U* tests performed on label-free quantification processed data. False discovery rate was controlled with the Benjamini–Krieger–Yekutieli procedure, and proteins with q < 0.05 (above the *dashed line*) were considered significant.

The filtered dataset comprised 334 proteins and protein groups (hereafter referred to as proteins in the results section). To establish a benchmark for the ML model, a Mann–Whitney *U* test was performed to identify differentially abundant proteins between combined AA and CRC groups (G2/G3) and the false-positive control group (G1) in the full dataset (141 samples). A total of 57 proteins exhibited a *p* value ≤0.05 (Fig. 2 and Supplemental Table S3); however, after multiple testing correction, 15 proteins with a q value <0.05 were retained. Manual inspection of precursor peak quality using Skyline revealed potentially unreliable quantification for two of these proteins (Supplemental Table S4), leading to their exclusion. This resulted in 13 significant proteins (Table 1).

**Table 1:** Differentially abundant proteins between G1 and G2/G3 groups

Protein group	First protein name	UniProt ID	*p* value	q value	Variation	PCA model
TAF2_HUMAN	Transcription initiation factor TFIID subunit 2	Q6P1X5	<0.001	<0.001	↑	Yes
CD44_HUMAN	CD44 antigen	P16070	<0.001	<0.001	↓	Yes
PG2IP_HUMAN	PGAP2-interacting protein	Q9H720	<0.001	0.018	↑	Yes
RIMS2_HUMAN	Regulating synaptic membrane exocytosis protein 2	Q9UQ26	<0.001	0.033	↓	Yes
IGLC7_HUMAN; IGLL5_HUMAN	Immunoglobulin lambda constant 7	A0M8Q6; B9A064	<0.001	0.034	↓	Yes
HBB_HUMAN	Hemoglobin subunit beta	P68871	<0.001	0.035	↑	Yes
HBB_HUMAN; HBD_HUMAN	Hemoglobin subunit beta;delta	P02042; P68871	<0.001	0.037	↑	No
KV116_HUMAN	Immunoglobulin kappa variable 1–16	P04430	0.001	0.037	↓	Yes
HBB_HUMAN; HBB_PIG; HBD_HUMAN	Hemoglobin subunit beta; delta	P02042; P02067; P68871	0.001	0.038	↑	No
APOD_HUMAN	Apolipoprotein D	P05090	0.001	0.039	↑	Yes
IGHA2_HUMAN	Immunoglobulin heavy constant alpha 2	P01877	0.002	0.039	↓	Yes
HBB_HUMAN; HBB_PIG	Hemoglobin subunit beta	P02067; P68871	0.002	0.039	↑	No
HBA_HUMAN	Hemoglobin subunit alpha	P69905	0.002	0.042	↑	No

A Mann–Whitney *U* test was applied to the label-free quantification dataset (334 proteins) to identify proteins differentially abundant between G1 and G2/G3. *p* values were adjusted for multiple testing using the Benjamini–Krieger–Yekutieli two-stage step-up method. After filtering for spectral evidence, 13 proteins with q values <0.05 were retained. The “variation” column indicates direction of change (↑ more abundant in G2/G3, ↓ less abundant in G2/G3). The “PCA model” column denotes whether the protein was included (yes or no) in the subsequent PCA-based model.

PCA, principal component analysis.

To establish a benchmark for ML classification performance (G2/3 *versus* G1), a PCA combined with a logistic regression model (PCA-based model) was trained on the full dataset. The PCA-based model was included as a baseline for comparison against more advanced supervised learning algorithms and was not intended to provide unbiased estimates of predictive performance.

PCA was initially performed on the set of 13 proteins (Table 1), including five hemoglobin-related protein groups. However, these hemoglobin proteins exerted a dominant influence on the first PC1, as reflected by their high PCA loading values (0.948–0.985). Given that the FIT test already measures hemoglobin, this strong contribution may not provide additional discriminative value so only the HBB protein was retained, resulting in a final selection of nine proteins for the PCA (Table 1). The first three PCs of this PCA were identified as significant, explaining a cumulative variance of 60.8% (PC1: 28.9%, PC2: 18.2%, PC3: 13.7%).

**Fig. 3:**
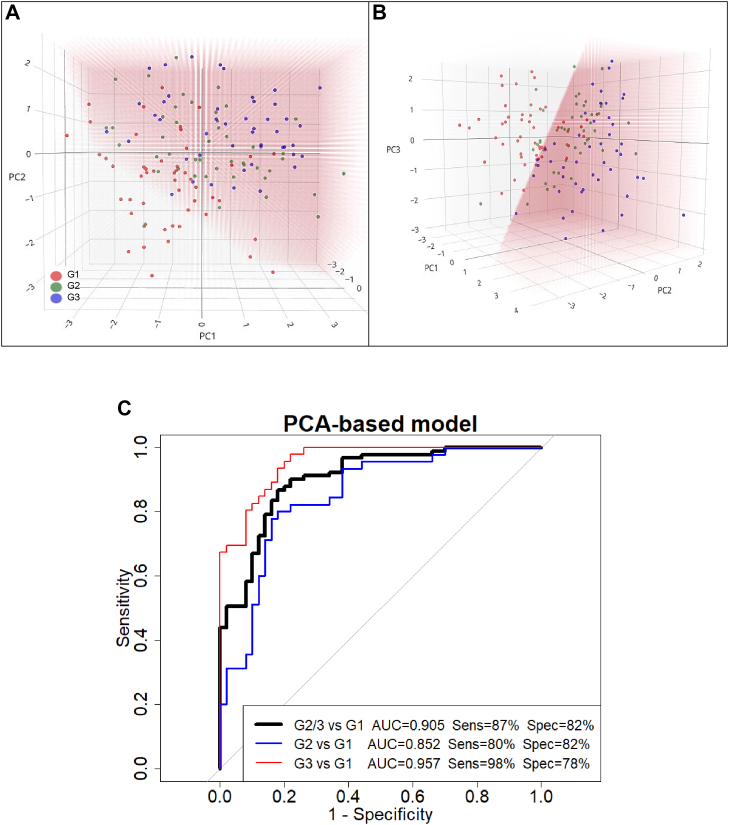
**PCA-based model for classification of G2/G3 *versus* G1 samples**. *A* and *B*, principal component analysis (PCA) was performed on nine differentially abundant proteins, followed by logistic regression on the three selected PCs to predict membership of the combined advanced adenoma (G2) and colorectal cancer (G3) group (G2/G3) *versus* false-positive controls (G1). *Panels A* and *B* show the same three-dimensional PCA plot from different viewing angles. The *pink-shaded background* indicates the logistic model’s linear classification boundary: clear regions correspond to predicted G1, while *red-shaded regions* correspond to predicted G2/G3. Samples are color-coded by actual class: G1 (*red*), G2 (*green*), and G3 (*blue*). *C*, ROC curves for the PCA-based model. The *black curve* represents G2/G3 *versus* G1, while *blue* and *red curves* represent G2 *versus* G1 and G3 *versus* G1, respectively. Higher AUROC for G3 *versus* G1 indicates stronger discriminative power for CRC. Optimal sensitivity and specificity values for each comparison were determined using the Youden index (95). AUROC, area under the receiver operating characteristic curve; CRC, colorectal cancer; PC, principal component; ROC, receiver operating characteristic.

A logistic regression model was then applied to the scores of these three PCs. Using a probability threshold of 0.5 for binary classification, this PCA-based model plot of the PCA scores shows how the samples distribute across the three PCs (Fig. 3, *A* and *B*). The model correctly classified 37 of 50 G1 samples (specificity: 74%) and 83 of 91 G2/3 samples (sensitivity: 92%), resulting in a balanced accuracy of 83%. Interestingly, analysis of G2 and G3 separately revealed that 37 out of 45 G2 samples (sensitivity: 82%) and all 46 G3 samples (sensitivity: 100%) were correctly classified.

The AUROC of 0.905 calculated from the predicted group membership probabilities, indicates that the model distinguishes samples from the G2/G3 group from the G1 control with an average accuracy of 90.5% across all classification thresholds (Fig. 3*C*, G2/3 *versus* G1). ROC curves computed separately for the G2 and G3 groups revealed that the model performed very well in discriminating CRC from control with an AUROC of 0.957 and relatively well in distinguishing AA from controls with an AUROC of 0.852 (Fig. 3*C*).

Overall, these metrics indicate that the PCA-based model achieved relatively strong predictive performance, confirming the presence of discriminative information in the dataset and providing a robust benchmark for subsequent comparisons with ML models.

### ML Feature Selection

The whole dataset was split into an 80% training set (113 samples: 40 G1, 73 G2/3 including 36 G2 and 37 G3) and a 20% held-out testing set (28 samples: 10 G1, 18 G2/3 including 9 G2 and 9 G3) for subsequent model validation. Considering the limited number of significant proteins based on q value <0.05, all the 334 proteins of the training dataset were used for the feature selection by five supervised ML algorithms (RF, RRF, Boruta, SVMSVM-RFE, Lasso).

**Fig. 4:**
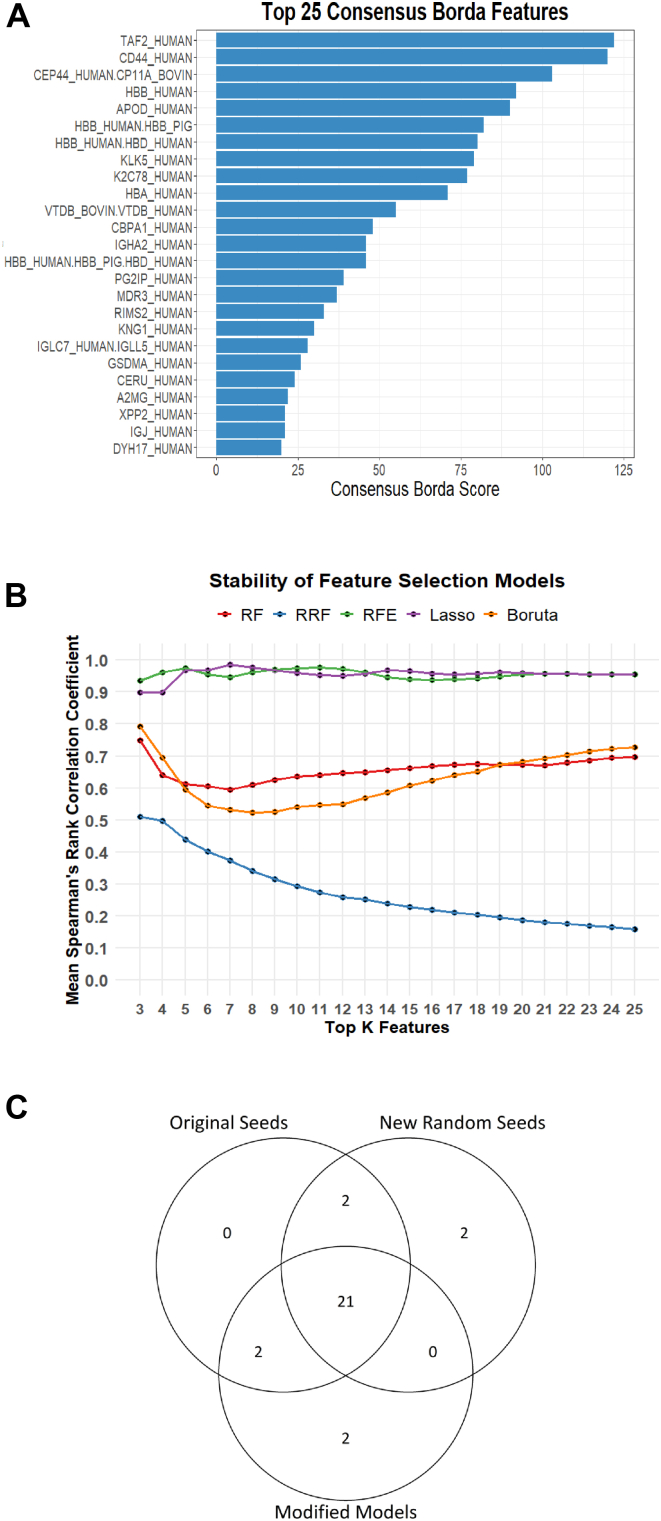
**Feature selection by machine learning models, stability and top 25 consensus features**. *A*, protein features distinguishing G1 and G2/G3 samples were ranked using five machine learning methods (Supplemental Fig. S1). The histogram shows consensus ranking of the top 25 features using Borda count integration across all methods. Features are ordered by decreasing Borda scores on the *y*-axis. *B*, mean pairwise Spearman rank correlation of selected features across 50 runs with varying random seeds, shown for each method: Random Forest (RF), Regularized Random Forest (RRF), Recursive Feature Elimination (RFE), Lasso, and Boruta. Spearman correlation coefficients were plotted for increasing top K features (3–25), with higher values indicating more consistent rankings. *C*, Venn diagram showing that 21 of the original top 25 consensus features (Original Seeds) remain consistent despite changes in random seed sets (New Random Seeds) and model configurations (Modified Models), with only minor differences (2 features) per condition, demonstrating the robustness of the Borda consensus approach.

ML models typically assess the predictive power of variables using importance scores, which reflect each variable’s influence on the model’s decision function. However, many ML models involve inherent randomness in data sampling and structural procedures, which can be reproduced by setting a fixed random seed (76). To ensure robust and consistent feature selection, each ML algorithm was executed 50 times with different random seeds. In each run, the top 25 features were ranked according to their feature importance scores. Then, Borda count–based rank aggregation was used to consolidate results across all runs and generate a consolidated feature ranking for each model (Supplemental Fig. S1, *A*–*E*). The 25 proteins with the highest cumulative Borda scores across all models were identified as the Top 25 consensus Borda features for further evaluation (Fig. 4*A*).

To assess models stability, feature importance ranking across the 50 random seeds was estimated using the mean pairwise Spearman’s rank correlation coefficient (range: −1 to 1), where higher values indicate stronger agreement in feature importance rank across runs (77, 78). Stability was evaluated across increasing subsets of top-ranked features, from the top three up to the top 25 features, thereby capturing how ranking agreement evolves as additional, lower-ranked features are included.

As shown in Figure 4*B*, Lasso and SVM-RFE exhibited consistently high stability across all subset sizes (Spearman ≈ 0.90–0.99), indicating highly reproducible feature rankings regardless of the number of top features considered. RF and Boruta showed moderate stability, with slightly lower agreement in the intermediate subset sizes (6–16), followed by a gradual increase in stability as the subset size expanded (Spearman K = 25 ≈ 0.72), suggesting convergence toward a more consistent feature ranking as additional features are included. In contrast, RRF demonstrated the lowest stability across all subset sizes, with stability decreasing as the number of included features increased (Spearman K = 25 = 0.16), indicating substantial variability in feature importance ranking across runs.

Despite the greater variability observed for the RRF model, the consensus Borda Top 25 feature list proved robust to both stochastic variation and modeling strategy. As shown in Figure 4*C*, changing the random seed preset series altered only two features (A2MG and XPP2). Likewise, modifying model configurations (such as using only confirmed Boruta features for importance ranking, adding bootstrapping to RRF and Lasso, and replacing SVM-RFE cross-validation with bootstrapping) resulted in differences in only two features (IGLC7 and IGJ). Together, these analyses demonstrate that the Borda count–based aggregation approach produces a reproducible and stable feature selection across multiple ML models.

### Features Downstream Filtering

Inspection of spectra in Skyline.ms revealed that five of the Top 25 consensus Borda features lacked sufficient evidence for consistent quantification across sample batches (Supplemental Table S4). These were excluded, leaving 20 features with at least one precursor showing consistent reliability across all batches. Notably, 12 of the 13 statistically significant proteins from Table 1 were present in this refined set.

Multiple hemoglobin protein groups were selected in the consensus list, suggesting high correlation among some features. To further reduce feature redundancy, multicollinearity among the selected proteins was assessed using the VIF. All the hemoglobin-related proteins had a VIF ≥10 (Supplemental Table S5*A*). Based on this analysis, four of the five hemoglobin-related proteins were excluded, prioritizing only the HBB protein for the benchmark model. Reanalysis of VIF confirmed that all the remaining features had a VIF score well below 10 (Supplemental Table S5*B*), resulting in a final panel of 16 features (Table 2).

**Table 2:** Final set of 16 features

Protein group	Abbr.	First protein name	UniProt ID	*p* value	q value	Variation
TAF2_HUMAN	TAF2	Transcription initiation factor TFIID subunit 2	Q6P1X5	**<0.001**	**<0.001**	↑
PG2IP_HUMAN	PG2IP	PGAP2-interacting protein	Q9H720	**<0.001**	**0.018**	↑
HBB_HUMAN	HBB	Hemoglobin subunit beta	P68871	**<0.001**	**0.035**	↑
APOD_HUMAN	APOD	Apolipoprotein D	P05090	**0.001**	**0.039**	↑
CERU_HUMAN	CERU	Ceruloplasmin	P00450	**0.014**	0.152	↑
GSDMA_HUMAN	GSDMA	Gasdermin-A	Q96QA5	**0.027**	0.226	↑
XPP2_HUMAN	XPP2	Xaa-Pro aminopeptidase 2	O43895	0.054	0.302	-
A2MG_HUMAN	A2MG	Alpha-2-macroglobulin	P01023	0.160	0.501	-
DYH17_HUMAN	DYH17	Dynein axonemal heavy chain 17	Q9UFH2	0.474	0.760	-
KNG1_HUMAN	KNG1	Isoform LMW of Kininogen-1	P01042	0.555	0.787	-
CBPA1_HUMAN	CBPA1	Carboxypeptidase A1	P15085	**0.017**	0.167	↓
IGJ_HUMAN	IGJ	Immunoglobulin J chain	P01591	**0.003**	0.053	↓
IGHA2_HUMAN	IGHA2	Immunoglobulin heavy constant alpha 2	P01877	**0.002**	**0.039**	↓
RIMS2_HUMAN	RIMS2	Reg. synaptic membrane exocytosis protein 2	Q9UQ26	**0.001**	**0.037**	↓
IGLC7_HUMAN; IGLL5_HUMAN	IGLC7	Immunoglobulin lambda constant 7	A0M8Q6; B9A064	**0.001**	**0.034**	↓
CD44_HUMAN	CD44	CD44 antigen	P16070	**< 0.001**	**0.006**	↓

This table lists the final 16 nonredundant protein features selected. The “variation” column indicates whether the protein is more (↑) or less (↓) abundant in G2/3 compared to G1, based on a *p* value ≤0.05.

Proteins with significant *p* values and q values are shown in bold.

Among the 16 selected features, 14 exhibited a *p* value ≤0.05 (Table 2), with eight overlapping with the previous list of nine significant proteins (q value <0.05) used for the previous PCA-based benchmark model. Conversely, three features (A2MG, DYH17, and KNG1) had a *p* value >0.05 but were retained (Table 2), as their selection by the ML model suggested these features might still provide information for group discrimination, even if they are not statistically significant on their own. The final 16-feature panel was thus established with minimal outlier impact.

### ML Model for Classification

Six supervised ML models (RF, XGBoost, KNNs, glmnet, SVM, and Naïve Bayes) were trained on the 80% training set to differentiate between the combined G2/3 groups and G1 group using the 16 ML-selected feature panel derived from the same dataset. Hyperparameter tuning was performed using cross-validation, with AUROC used as the primary metric for selecting optimal model configurations (Supplemental Table S2).

**Fig. 5:**
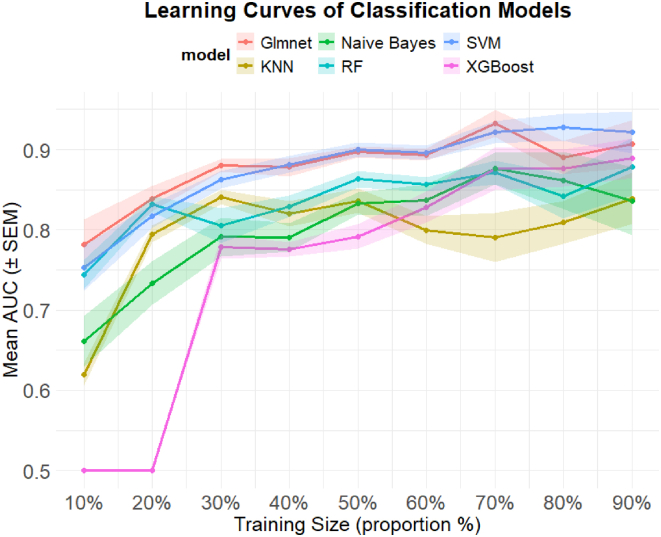
**Hybrid learning curves of the supervised ML classification models**. Mean AUROC (±SEM) is shown as a function of training set proportion (10–90%) for six supervised machine learning models evaluated using repeated stratified subsampling of the training dataset. Models were retrained at each training size using previously identified optimal hyperparameters and evaluated on the corresponding internal hold-out sets (90–10%). *Shaded regions* represent the SEM across repeats. ML, machine learning; AUROC, area under the receiver operating characteristic curve.

To evaluate the learning behavior of the models, a hybrid learning-curve analysis was conducted using training subsets representing 10 to 90% of the original 80% training set (see Model Evaluation and Interpretability in the Procedures section for details). Hyperparameters were fixed at their optimal configuration values (as determined above) to ensure that the learning curves reflected only the effect of training-set size and not of repeated hyperparameter tuning. Performance at each training size was evaluated on the corresponding held-out subsets (90%–10%) and plotted as mean AUROC ± SEM across subsampling iterations to visualize learning dynamics and model stability. As shown in Figure 5, glmnet and SVM consistently outperformed the other models across most training sizes and were the only two methods to exceed an AUROC of 0.9 at higher training subset proportions. SEM values for these two models remained low, with a slight increase at 80 to 90%, likely reflecting increased variability associated with smaller hold-out sample sizes. The progressive flattening of their learning curves with increasing training subset size suggests that these models captured most of the predictive signal available in the data. In contrast, XGBoost, the third-best performing model at 90% training size, displayed a continuously rising curve, suggesting that additional training data could potentially further improve its performance. Overall, glmnet and SVM demonstrated more favorable learning dynamics than the other models, with glmnet achieving slightly higher AUROC at smaller training sizes and SVM performing slightly better at the largest training sizes, albeit within overlapping SEM bounds.

Guided by the learning curve analysis, we evaluated the performance of the fitted models using predicted probabilities from the 80% training set. As summarized in Table 3, five of the six models achieved higher AUROC values than the previously reported PCA-based benchmark model (Fig. 3), indicating improved discriminatory power with supervised learning approaches. However, comparison of training AUROC results with mean CV AUROC values revealed that RF, XGBoost, Naïve Bayes, and KNN exhibited relatively large positive differences (ΔAUROC), suggesting greater susceptibility to overfitting. In contrast, glmnet and SVM showed minimal discrepancies between training and CV performance (Table 3: <5%), indicating more stable generalization behavior (69).

**Table 3:** Performance metrics of machine learning models

ML model	Data	Sensitivity	Specificity	BA	AUROC	CI (95%)	CV AUROC fold mean ± SD	ΔAUROC (training - CV)	ΔAUROC/CV AUROC (%)
RF	Train	1.000	0.975	0.988	1.000	1.000–1.000	0.904 ± 0.088	0.096	10.6%
XGBoost	Train	0.986	0.950	0.968	0.998	0.993–1.000	0.882 ± 0.096	0.116	13.1%
Naïve Bayes	Train	0.932	0.675	0.803	0.938	0.893–0.983	0.851 ± 0.117	0.087	10.2%
KNN	Train	0.945	0.475	0.710	0.866	0.800–0.933	0.799 ± 0.144	0.067	8.4%
Glmet	Train	0.904	0.800	0.852	0.956	0.924–0.989	0.921 ± 0.076	**0.035**	**3.8%**
Glmet	Test	0.889	0.800	0.844	0.961	0.897–1.000	—		
SVM	Train	0.918	0.825	0.871	0.968	0.942–0.993	0.927 ± 0.068	**0.035**	**4.4%**
SVM	Test	0.889	0.800	0.844	0.950	0.877–1.000	—	—	

This table summarizes the performance of final fitted classification models for both the training set (80% of samples: all models) and the testing/validation set (20% held-out samples: glmnet and SVM). Reported metrics include sensitivity, specificity, balanced accuracy (BA) using a probability threshold of 0.5 for binary classification, and AUROC, with 95% confidence intervals (CIs). Cross-validation (CV) mean and SD of AUROC across folds, along with absolute and percentage differences between model classification and CV AUROC, are also included to evaluate potential overfitting. Bold text is for the the two selected models.

AUROC, area under the receiver operating characteristic curve; ML, machine learning; SVM, Support Vector Machine.

Consequently, both models were selected for evaluation of their generalization on the independent on the independent 20% held-out testing set, where each demonstrated consistent classification performance relative to the 80% training set (Table 3). Taken together, these findings identify glmnet and SVM as the most robust models in this study, combining favorable learning dynamics with limited overfitting potential and strong generalization. Given their comparable performance, glmnet was selected as the final model due to its lower complexity and greater interpretability, making it more suitable for biological interpretation.

**Fig. 6:**
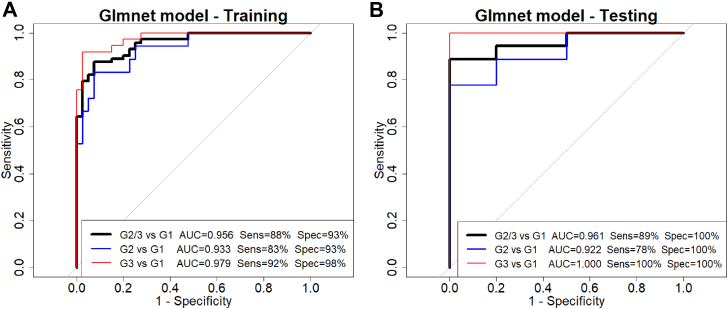
**ROC curves for model performance evaluation**. Panels (*A* and *B*) show ROC curves of the fitted glmnet model obtained from (*A*) the 80% training set and (*B*) the 20% held-out testing set for G2/G3 *versus* G1 (*black*), G2 *versus* G1 (*blue*), and G3 *versus* G1 (*red*) subsets. Corresponding AUROC values indicate model performance. The figure also reports the corresponding optimal sensitivity and specificity values for each comparison, determined using Youden-optimal threshold (95). AUROC, area under the receiver operating characteristic curve; ROC, receiver operating characteristic.

Finally, ROC curves for the fitted glmnet model were generated separately for the G2 (AA) and G3 (CRC) subgroups versus the G1 (false-positive control) for the results of the 80% training set and the 20% held-out testing set. These analyses confirmed strong classification performance across subgroups and datasets (Fig. 6, *A*–*B*), with higher performance observed for CRC samples. Collectively, this supports the potential of glmnet as a reliable classifier for colorectal precancerous lesions and cancer in screening applications.

### SHAP Analysis of Feature Contributions in Glmnet Model

The feature contribution to the fitted glmnet model was evaluated using SHAP analysis which calculates the average marginal contribution of a feature value across all possible feature combinations, attributing to each feature its expected impact on the model's class prediction output.

**Fig. 7:**
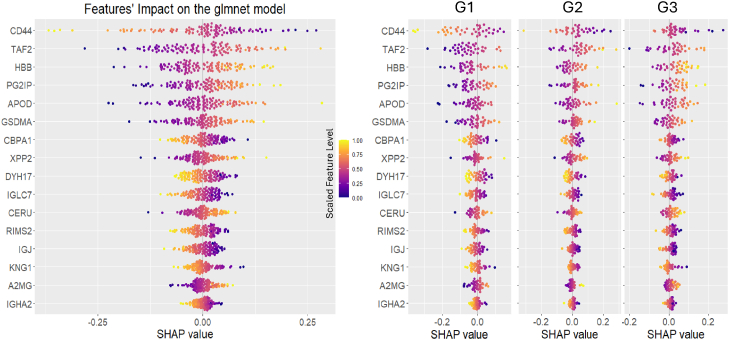
**SHAP analysis of glmnet model feature contributions**. Beeswarm plots showing individual the feature impacts on glmnet model predictions via SHAP values. Each point represents a sample; the *x*-axis shows the SHAP value, reflecting the feature’s influence on predicting the target class (G2/G3). Positive values increase the likelihood of G2/G3 classification, while negative values decrease it. The *left panel* displays the global SHAP distribution model, while the *right panels* show the corresponding distributions stratified by group (G1, G2, and G3). The color gradient legend represents the feature abundance level for each sample, scaled from 0 (*dark blue*) to 1 (*yellow*). SHAP, SHapley Additive exPlanations.

While Table 4 quantifies the feature impact on the glmnet model, the beeswarm plots (Fig. 7) illustrate how these impacts vary across different samples and groups. CD44 and TAF2 emerged as the most influential features highlighting their potential as key biomarkers in this study. On the other hand, KNG1, A2MG and IGHA2 exhibited the lowest mean absolute SHAP values (≤0.02), indicating less influence on model predictions.

**Table 4:** SHAP values for feature importance in glmnet model

Glmnet model		
Feature	Mean Abs. SHAP	SD SHAP
CD44	0.083	0.116
TAF2	0.077	0.097
HBB	0.061	0.074
PG2IP	0.055	0.069
APOD	0.051	0.071
GSDMA	0.041	0.054
CBPA1	0.031	0.039
XPP2	0.031	0.042
DYH17	0.029	0.035
IGLC7	0.026	0.033
CERU	0.025	0.033
RIMS2	0.024	0.030
IGJ	0.023	0.028
KNG1	0.020	0.028
A2MG	0.020	0.027
IGHA2	0.015	0.018

Mean absolute SHAP values (mean Abs. SHAP) and their SDs (SD SHAP) are shown for 16 features, ranked by importance for the glmnet model. Higher mean Abs. SHAP indicates greater feature impact; SD SHAP reflects variability across samples.

SHAP, SHapley Additive exPlanations.

## Discussion

In the context of a study aimed at evaluating the potential of proteomics for identifying false-positive patients in colorectal cancer FIT-based screening to prevent unnecessary colonoscopies, predictive strategies were tested to distinguish AA and CRC from control samples based on label-free quantitative proteomic data derived from SWATH analysis of residual FIT samples. The results show that both PCA-based regression model and ML-based feature selection led to reliable identification of controls, AA and CRC patients with high accuracy.

As a benchmark model the overall PCA-based regression approach should be considered supervised, as the logistic regression is explicitly trained to classify samples into their respective class by defining a linear decision boundary in PC space. Although the regression model could have been applied directly to the nine differentially abundant features, unsupervised PCA was used to reduce dimensionality, mitigate potential multicollinearity and noise, while preserving the most informative variance structure in the data (41). Importantly, this PCA-based benchmark was trained on the full sample dataset without independent evaluation of generalization performance. Consequently, its performance should be interpreted as an optimistic reference rather than a deployable predictive model. Despite its relative simplicity and limitations, this model demonstrated very good discrimination, particularly for the classification between CRC and control samples, implying that the protein abundance changes in CRC samples are sufficiently pronounced to be reliably identified by a relatively straightforward approach. AA samples were slightly more difficult to classify correctly, possibly reflecting more subtle proteomic alterations associated with early-stage carcinogenesis (79), an observation that aligns with the adenoma miss rate at colonoscopy (80).

In contrast to the PCA-based model, the supervised ML portion of this study followed a conventional 80% training/20% held-out testing framework to enable unbiased evaluation of generalization performance. The ML-based feature selection pipeline applied five distinct methods on the 80% training dataset to capture both linear and nonlinear patterns that are not detectable through univariate statistical analyses. However, without prior filtering of correlated features, redundancy can cause instability in ML feature importance scoring and selection (77). To address this, the robustness of feature selection was enhanced by employing a random seed–based resampling strategy combined with Borda count rank aggregation. Overall, this strategy appeared particularly beneficial for decision tree–based models, namely RF, Boruta, and RRF, which showed varying levels of stability. RF and Boruta (an extension of RF that uses permutation tests and shadow features to identify all relevant variables) (81) showed moderate stability. This variability is consistent with the inherent randomness of RF-based methods, which rely on bootstrapped sampling and random feature selection at each split, factors known to introduce variability in feature importance measures (82). Conversely, RRF, which builds trees sequentially using a regularization strategy to penalize redundant or weak predictors (83, 84), demonstrated greater instability. This likely reflects RRF’s sensitivity to multicollinearity and stochastic elements in tree construction, such as sample selection and split criteria, making its feature rankings more susceptible to variability. Consequently, aggregating feature importance across multiple runs helped achieve more reproducible rankings for these tree-based models. In contrast, for more deterministic models like Lasso and SVM-RFE, aggregation was likely less critical. These models, combined with internal cross-validation, already showed more stable feature selection, which contributed to the consistency of predictor subsets across runs. Additionally, both Lasso and SVM-RFE inherently tend to select smaller feature subsets (85, 86).

Downstream filtering to remove features with inconsistent signals and multicollinearity generated a biologically plausible panel of 16 proteins, which were then used to train six supervised ML classification algorithms—RF, glmnet, XGBoost, KNN, SVM, and Naïve Bayes. The 80% training dataset comprised 113 samples, resulting in approximately seven samples per feature, with an events-per-variable ratio of 4.6 (73 G2/3-positive cases and 16 predictors). Although this is below the commonly recommended threshold of ≥10:1 (77), the risk of overfitting was mitigated through preselection of features to reduce redundancy and the use of repeated stratified cross-validation. This cross-validation strategy also addressed the moderate class imbalance (G1: 40 samples; G2/3: 73 samples) by maintaining proportional class representation in each fold, thereby enhancing the robustness and fairness of performance estimates (66). Additionally, stratification was applied during the train/test dataset split to ensure consistent class distribution across subsets and prevent dataset shift, further supporting unbiased evaluation on the independent testing subset.

Even though sensitivity and specificity were evaluated using uncalibrated predicted probabilities, the primary evaluation metric was AUROC, which remains unaffected by probability scaling and class proportions. This makes AUROC particularly suitable for moderately imbalanced datasets (66). Furthermore, AUROC reflects the model's ability to rank positive instances above negatives, a critical property in clinical screening contexts where identifying high-risk individuals is a priority. Among the 6 ML classifiers evaluated, the fitted glmnet and SVM models demonstrated the most consistent and generalizable performance across all evaluation criteria. Both models achieved high AUROC values on the 80% training set, with cross-validation AUROC within 5%, and maintained similarly strong performance on the 20% held-out testing set, indicating limited overfitting. The hybrid learning curve analysis also supported these findings, suggesting efficient use of the available data and stable generalization with the two models. Interestingly, AUROC values from the testing set slightly exceeded those estimated from the learning curve at the highest training size proportion, which likely reflects minor sampling variability and suggests that the testing set may have been somewhat easier to classify. This also suggests that the use of nested cross-validation might have provided a more conservative estimate of the ML model performance from the 80% training dataset, therefore reducing the risk of overestimating the classification metrics during model evaluation.

Ultimately, given the comparable predictive performance of the glmnet and SVM models, the glmnet model was favored due to its greater interpretability. Its robust performance under limited sample size conditions can be attributed, at least in part, to its regularized linear framework, which typically requires fewer events per variable than more complex nonlinear classifiers (67). The optimal hyperparameters corresponded to a ridge (L2) penalty (α = 0) with moderate regularization strength (λ = 0.06) (56). This configuration stabilizes coefficient estimates by shrinking correlated feature weights toward each other rather than enforcing sparsity, thereby effectively managing multicollinearity while preserving the contribution of informative but correlated proteins (56). For instance, among our panel of 16 features, a subset of immunoglobulin-related features (IGHA2, IGJ, and IGLC7; VIF ≈ 4–6) was retained despite moderate multicollinearity. These features ranked among the lowest in mean absolute SHAP values, with IGHA2 being the least influential predictor overall—a pattern consistent with ridge regularization, distributing predictive responsibility across correlated features.

While all ML classifiers could benefit from larger sample sizes, since regularization alone cannot fully compensate for low event-per-variable ratios (87), the glmnet model nevertheless excelled at predicting CRC samples and overall achieved higher AUROC values than the PCA-based benchmark model. However, comparisons of these AUROC values across all predictions (training + testing) and models, using DeLong’s test for correlated ROC curves (88), revealed no statistically significant differences in performance for either the group-specific (G2 *versus* G1 and G3 *versus* G1) or combined group (G2/G3 *versus* G1) comparisons after Benjamini–Hochberg correction for multiple testing (*p* > 0.05; results not shown).

Potentially interesting interactions among the selected features were noted across biological processes, molecular functions and cellular components. Notably, A2MG and IGHA2 were consistently implicated in several immune- and inflammation-related pathway, including cytokine binding and the negative regulation of complement activation, lectin pathway, which is a crucial part of the innate immune system activated by pathogens (89). Additional inflammation-associated pathways involving A2M, IGHA2, and KNG1 included endopeptidase inhibitor activity and platelet alpha granule lumen, a specialized platelet secretory organelle involved in coagulation, inflammation, angiogenesis, and wound healing (90). Additionally, immune-related processes such as humoral immune response and IgA immunoglobulin complexes were linked to IGHA2, IGLC7, and IGJ. Other proteins such as HBB, KNG1, CERU, XPP2, PG2IP, and APOD were found to be involved in blood vessel diameter maintenance, peroxidase activity, metalloexopeptidase activity, and regulation of protein import into the nucleus. Altogether, these results point to a functional environment enriched in immune and inflammatory responses, as well as vascular and tissue homeostasis processes, which are also relevant to tissue remodeling during cancer progression.

The potential impact of this study for the process of colorectal cancer screening in the clinic is at multiple levels. The major added value in the screening process by the introduction of an intermediary test between FIT and colonoscopy would be a significant reduction of the false FIT positives required to undertake the endoscopy procedure, which may represent up to 60% of all FIT positives (29, 30). Three main outcomes of reducing false FIT positives are predictable: (1) Substantial reduction of the total number of colonoscopies would represent a major economy to both the health system and patients. Indeed, colonoscopy is a relatively expensive clinical modality while the burden of health expenses is out of control in most countries. Time spent for the preparation and the procedure has also non-negligible financial impacts on the patients. (2) A corollary of the reduction of false FIT positives would be an increase in confidence for the need to agree to undertake a colonoscopy. At this time, it appears that a significant proportion of the patients identified as FIT positive do not further the screening process for CRC toward colonoscopy (91). Fewer false FIT positives resulting from the intermediate test may become a strong argument for convincing patients to continue the screening procedure up to the colonoscopy thus increasing adherence. (3) Another significant outcome resulting from the reduction of the clinical burden in endoscopy centers resulting from the reduction of colonoscopies from false positives would be to diminish the screening age to 45 years as recommended (15) as well as reducing the cutoff (92).

A successful implementation of such intermediate modality to reduce false FIT-positive tests would imply that a minimum of patients with AA or CRC would be exempted from a colonoscopy. Indeed, in a clinical context, it is crucial that patients with CRC and a good proportion of patients with AA be selected for colonoscopy while reducing the number of false-positive patients that need to undergo colonoscopy. Using fixed specificity to 80%, which concedes 20% of false positives that will be recommended for colonoscopy, sensitivity reaches 98% for CRC detection and 82% for AA detection, confirming the robustness of this intermediate triage test. These results indicate that the glmnet model reliably identifies discriminative protein signatures, delivering clinically relevant predictive power for FIT residual sample screening, with optimal balance between sensitivity and specificity. Further studies using quantitative targeted MS on stool samples are required for confirmation, but the current work demonstrates that the goal is achievable with this approach. As an outcome of the fact that CRC and AA can be differentiated in the proteomic stool-based test, it would be possible to prioritize patients with CRC for a fast-track colonoscopy, considering that a delay in colonoscopy after positive FIT test leads to higher incidence of colorectal cancer (93).

Finally, an additional strength of this intermediate test based on using the same sample as the one used for FIT is that most, if not all, recent versions of the screening tests for CRC have an arm testing occult blood in the stools (23, 24, 26, 27, 28).

The main limitation of the present study is that it is a first study of its kind. While the question of whether it is possible to use the remains of the FIT test to reanalyze positive samples for proteomics signatures has been successfully answered here, a number of validation steps are still mandatory before a clinically reliable test becomes available. Indeed, as mentioned above, the setup of a quantitative targeted MS analysis on stool samples is required for testing on larger and prospective cohorts of people from distinct origins.

In conclusion, in this study, we have explored the potential of proteomics combined with AI- and ML-based analyses for further analyzing FIT-positive samples as an intermediate step before colonoscopy for CRC screening. The identification of a large proportion of the false FIT-positive samples is an efficient means of reducing unnecessary colonoscopies, while the precise prediction of CRC among true FIT-positive samples may contribute to prioritizing the selection of patients for colonoscopy.

## Data Availability

The mass spectrometry proteomics data have been deposited to the ProteomeXchange Consortium via the PRIDE (94) partner repository (http://www.ebi.ac.uk/pride, accessed on July 31, 2025) with the dataset identified PXD066834.

## Supplemental Data

This article contains supplemental data.

## Conflictof Interest

H. G. and J.-F. N. are the CSO & Director and employee, respectively, of Allumiqs. D. G., E. S., M. M., H. G., J.-F. N., and J.-F. B. are the inventors of the intellectual property owned by TransferTech Sherbrooke, a valorization society for the Université de Sherbrooke, and the subject of a provisional patent. The other authors declare no competing interests.
