# Supplementary material for: Exploring an Intermediate Colorectal Cancer Screening Test Based on Stool Proteomics and Machine Learning for Optimizing the Selection of Patients for Colonoscopy Identified From FIT
Source: Mol Cell Proteomics. 2026 Feb 19;25(4):101534. doi: 10.1016/j.mcpro.2026.101534 (PMC13053997; doi:10.1016/j.mcpro.2026.101534)
Supplement: Supplemental Materials [file mmc1.pdf]

Supplemental data

Exploring an Intermediate Colorectal Cancer Screening Test Based on Stool Proteomics and Machine Learning for Optimizing the Selection of Patients for Colonoscopy Identified from FIT

David Gagné[1,2,3], Elmira Shajari[1,2,3], Mandy Malick[1,2,3], Patricia Roy[1.2.3], Jean-François Noël[4], Hugo Gagnon[4], Marie A. Brunet[2,5], Julie C. Carrier[2,6], François-Michel Boisvert[2,3], Jean-François Beaulieu[1,2,3]*

Page S2: Figure S1

Page S3: Table S2

Page S4: Table S4

Page S5: Table S5

Please note that Table S1 and Table S3 are provided in separate Excel files.

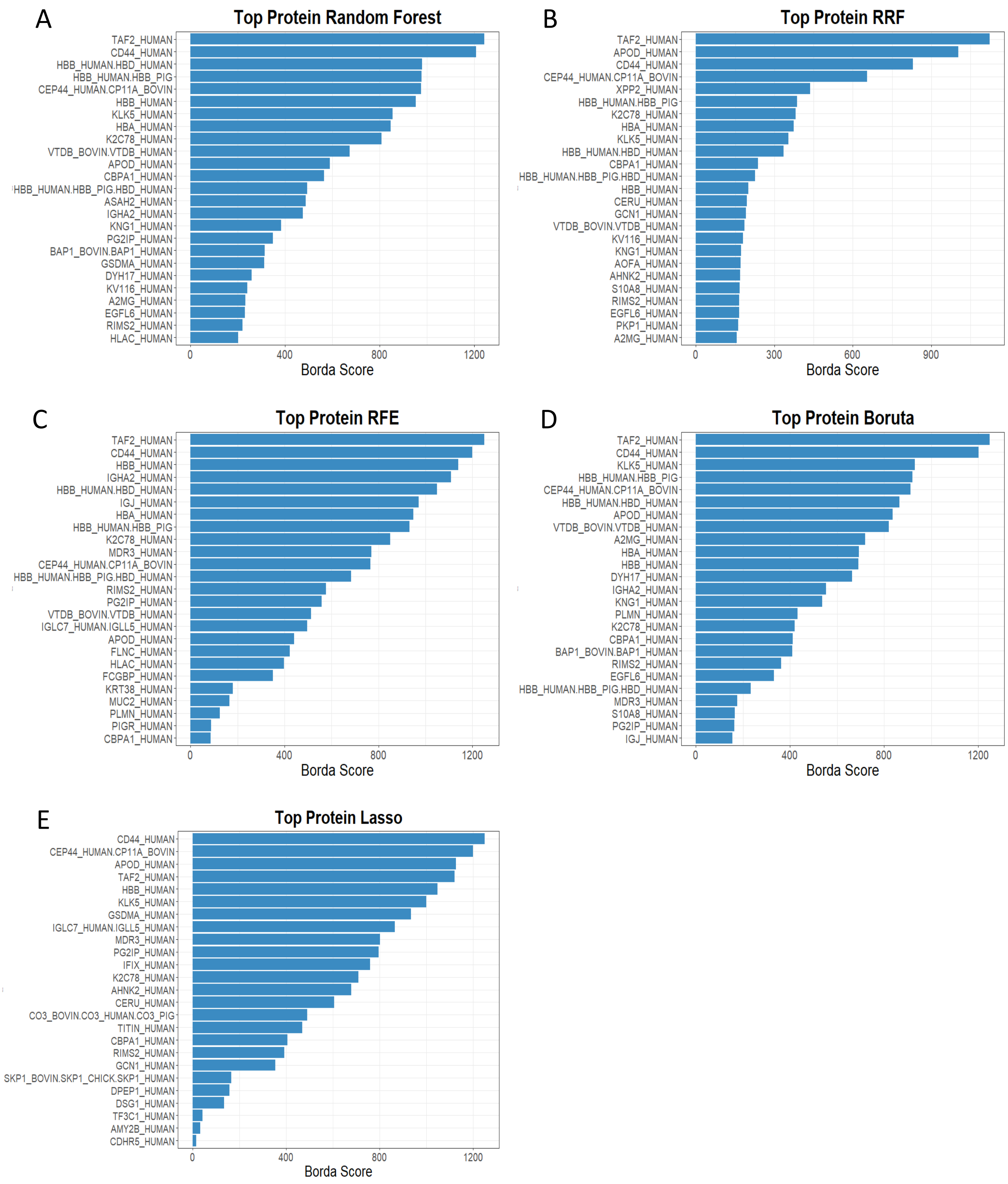

Figure S1. Feature Selection by Machine Learning Models. Protein features distinguishing G1 and G2/3 samples were ranked using five machine learning methods. (A–E) Borda-ranked importance scores after 50 runs with different random seeds from Random Forest, Regularized Random Forest (RRF), Recursive Feature Elimination (RFE), Boruta, and Lasso.

Table S2. Machine Learning Classification Models Hyperparameter Search Space

Model (method)	Hyperparameters search grid (Cross-validation selected parameters in **bold**)
Random Forest (ranger)	mtry: {2, 4, **6**, 8}; splitrule: {"gini","**extratrees**"}; min.node.size: {1, 2, **3**, 4, 5, 10}
XGBoost (xgbTree)	nrounds: {50, **75**, 100}; eta: {**0.05**, 0.1}; max_depth: {2, **3**}; gamma: {0.5, **1**}; colsample_bytree: {**0.6**, 0.8}; min_child_weight: {**3**, 5}; subsample: {0.6, **0.8**}
Naïve Bayes (nb)	usekernel: {**TRUE**, FALSE}; adjust: {0.2–3, step by = 0.2} = **2.6**; fL (Laplace): {1–3, step by = 0.1} =**1**
K-Nearest Neighbors (KNN)	k_values_for_nearest_neighbors : {3, 5, 7, 9, **11**}
Penalized Logistic Regression (glmnet)	alpha: {0–1, step by = 0.1} = **0**; lambda: {10 ^ seq(-5, 0, length.out = 30)} = **0.0621**
SVM (svmPoly)	degree: {1, **2**, 3}; scale: {2 ^ seq(-8, 2, by = 1)} = **0.0312**; C: {2 ^ seq(-7, 6, by = 1)} = **0.5**

Table S4. Protein groups removed prior to multivariate analysis, along with the rationale for exclusion.

Removed Protein group (Model)	Reason (PCA/ML)
MDR3_HUMAN (PCA, ML)	Low spectral evidence/possible interference
VTDB_BOVIN;VTDB_HUMAN (PCA, ML)	Low spectral evidence/possible interference
CEP44_HUMAN;CP11A_BOVIN (ML)	Low spectral evidence/possible interference
KLK5_HUMAN (ML)	Low spectral evidence/possible interference
K2C78_HUMAN (ML)	Low spectral evidence/possible interference
HBB_HUMAN;HBB_PIG (PCA, ML)	Dominant influence on PC1/High VIF
HBB_HUMAN;HBD_HUMAN (PCA, ML)	Dominant influence on PC1/High VIF
HBA_HUMAN (PCA, ML)	Dominant influence on PC1/High VIF
HBB_HUMAN;HBB_PIG;HBD_HUMAN (PCA, ML)	Dominant influence on PC1/High VIF

Removals were based on low spectral evidence/possible interference or excessive influence on model behavior. PCA = principal component analysis; ML = machine learning; VIF = variance inflation factor.

Tables S5A and S5B. Evaluation of multicollinearity among selected protein features using the Variance Inflation Factor (VIF) metric

A

Feature	VIF
HBB_HUMAN	33.2
HBA_HUMAN	29.0
HBB_HUMAN.HBB_PIG	28.8
HBB_HUMAN.HBD_HUMAN	20.8
HBB_HUMAN.HBB_PIG.HBD_HUMAN	9.9
IGHA2_HUMAN	6.0
IGJ_HUMAN	4.9
IGLC7_HUMAN.IGLL5_HUMAN	4.5
RIMS2_HUMAN	3.4
DYH17_HUMAN	2.7
KNG1_HUMAN	1.9
CERU_HUMAN	1.7
APOD_HUMAN	1.6
PG2IP_HUMAN	1.5
CBPA1_HUMAN	1.5
TAF2_HUMAN	1.4
GSDMA_HUMAN	1.4
CD44_HUMAN	1.3
A2MG_HUMAN	1.3
XPP2_HUMAN	1.2

B

Feature	VIF
IGHA2_HUMAN	5.6
IGJ_HUMAN	4.8
IGLC7_HUMAN.IGLL5_HUMAN	4.3
RIMS2_HUMAN	3.4
DYH17_HUMAN	2.2
KNG1_HUMAN	1.8
APOD_HUMAN	1.5
CERU_HUMAN	1.5
HBB_HUMAN	1.5
CBPA1_HUMAN	1.4
PG2IP_HUMAN	1.4
GSDMA_HUMAN	1.3
TAF2_HUMAN	1.3
A2MG_HUMAN	1.3
CD44_HUMAN	1.3
XPP2_HUMAN	1.1

To reduce redundancy, VIF was calculated, with values ≥10 considered indicative of strong multicollinearity. (S5A) All hemoglobin-related proteins exhibited high VIF scores, leading to the exclusion of four out of five such features, while retaining only HBB_HUMAN for downstream analysis. (S5B) Reanalysis after removal confirmed that the remaining 16 features had VIF scores well below the threshold, supporting their inclusion in the final feature panel.
